# No Evidence of the Ego-Depletion Effect across Task Characteristics and Individual Differences: A Pre-Registered Study

**DOI:** 10.1371/journal.pone.0147770

**Published:** 2016-02-10

**Authors:** John H. Lurquin, Laura E. Michaelson, Jane E. Barker, Daniel E. Gustavson, Claudia C. von Bastian, Nicholas P. Carruth, Akira Miyake

**Affiliations:** Department of Psychology and Neuroscience, University of Colorado Boulder, Boulder, Colorado, United States of America; Tilburg University, NETHERLANDS

## Abstract

Ego-depletion, a psychological phenomenon in which participants are less able to engage in self-control after prior exertion of self-control, has become widely popular in the scientific community as well as in the media. However, considerable debate exists among researchers as to the nature of the ego-depletion effect, and growing evidence suggests the effect may not be as strong or robust as the extant literature suggests. We examined the robustness of the ego-depletion effect and aimed to maximize the likelihood of detecting the effect by using one of the most widely used depletion tasks (video-viewing attention control task) and by considering task characteristics and individual differences that potentially moderate the effect. We also sought to make our research plan transparent by pre-registering our hypotheses, procedure, and planned analyses prior to data collection. Contrary to the ego-depletion hypothesis, participants in the depletion condition did not perform worse than control participants on the subsequent self-control task, even after considering moderator variables. These findings add to a growing body of evidence suggesting ego-depletion is not a reliable phenomenon, though more research is needed that uses large sample sizes, considers moderator variables, and pre-registers prior to data collection.

## Introduction

The idea that self-control is like a muscle—temporarily weakened following exertion, but strengthened with practice over time—is an elegant analogy that has grown increasingly popular over the past 15 years or so. This so-called “strength model of self-control” (for a review, see [1]) posits that engaging in self-control (e.g., overriding prepotent responses, ignoring distracting stimuli, making choices) draws from an internal bank of limited self-control resources. Performing an act of self-control diminishes these resources, and thereby reduces the ability to effectively engage in any subsequent act of self-control, a state termed “ego-depletion.”

Support for this ego-depletion effect stems primarily from experiments using the sequential task paradigm (e.g., [2]). In this paradigm, participants are first randomly assigned either to an ego-depletion condition in which they perform a self-control task (e.g., suppressing facial expressions, performing the incongruent trials of a Stroop task) or to a corresponding control condition in which they perform a task assumed to require less self-regulatory effort (e.g., freely expressing emotions, performing the neutral or congruent trials of the Stroop task). After the initial task, all participants perform a different task (often referred to as the outcome task) assumed to also rely on self-control. Worse performance on the outcome task by depletion participants compared to control participants is interpreted as evidence for the ego-depletion phenomenon and for the strength model of self-control.

Since the initial demonstration of this phenomenon [2], the ego-depletion effect has been demonstrated across a wide array of domains such as decision-making [3], social rejection [4], and executive functioning [5], and thus appears to be a robust and reliable phenomenon. Indeed, a meta-analysis of 83 ego-depletion articles reported that although some published studies failed to replicate the phenomenon, the vast majority of published studies demonstrate evidence consistent with ego-depletion, with a large average effect size of 0.62 (Cohen’s *d*; [6]). Although there are some recent attempts to develop theoretical alternatives to the strength model (e.g., [7–8]), even these models seem to be based on the assumption that ego-depletion is a robust phenomenon that must be explained by empirically-grounded theories of self-control.

Despite such prior evidence reported in the literature, some recently published articles have started to cast doubt on not only the magnitude and robustness of the effect but even the very existence of the ego-depletion effect, as will be reviewed shortly. In light of recent controversies surrounding the replicability of some well-known social psychological phenomena, such as behavioral priming [9–11], it seems highly important to rigorously examine how reliable and robust the ego-depletion effect actually is. The present study contributes to such an effort by reporting one pre-registered study that tested a set of a priori hypotheses about the ego-depletion effect and several variables that may potentially moderate the magnitude of the effect.

### Is the Ego-Depletion Effect Robust and Reliable?

There are a number of reasons why reexamining the robustness of the ego-depletion effect is needed, despite Hagger et al.’s [6] meta-analytic study reporting a strong average effect size (Cohen’s *d* = .62). Here, we review several reasons.

#### Publication bias

In the Hagger et al. [6] meta-analysis, 198 individual experiments were analyzed, but, of those, 47 experiments did not show statistically significant results. There are also more recent failures to replicate the ego-depletion effect [12–13]. The question is how common such replication failures are and how many additional replication failures are in the “file drawer,” given the well-known publication bias (i.e., the reluctance of researchers to submit and for journals to publish null findings). The rate of confirmed hypotheses in published psychology studies is estimated at 92% [14], which is much higher than should be expected given typical effect sizes and statistical power. This points to the strong likelihood of selective reporting of confirmative and significant results [15], leading small or unreliable effects to appear larger and more reliable than they actually are, and possibly even cultivating the illusion that a phenomenon exists when it actually does not [16].

Emerging data-analytic techniques have recently been applied to the ego-depletion literature to assess the credibility of this body of research. One technique, the “incredibility index” (IC index) [17], computes the probability that the set of studies contains fewer non-significant findings than would be credible given their power. Carter and McCullough [18] applied this technique to the studies included in the Hagger et al. [6] meta-analysis. A post-hoc power analysis of the studies estimated average power to be 0.55, which resulted in an IC-index greater than 0.999. That is, there is a 99% probability that the studies included in the Hagger et al. [6] meta-analysis were pulled from a larger body of research, much of it unpublished, that included more null and negative results than the 47 (out of 151) originally reported.

#### Small-study effects

Another related reason for reexamining the ego-depletion effect is that the majority of studies employed only small samples. Effect sizes from small samples are highly variable (i.e., large standard errors), which will lead to occasionally inflated effect sizes. Small sample sizes are especially problematic in the presence of publication bias. For example, assuming a true effect of zero, small studies would result in both positive and negative effects of highly variable magnitude. However, given the publication bias against reporting non-significant findings and findings that contradict a prevailing theory, only the strong positive effects are made known to the scientific community. In this example, publication bias leads to small study effects (i.e., smaller studies systematically result in different effect sizes than larger studies).

Carter and McCullough [18] estimated the influence of small-study effects in the ego-depletion literature by examining the correlation between the magnitude of effect sizes and their standard errors for the studies included in the Hagger et al. [6] meta-analysis, resulting in a significant positive correlation. The majority of previous ego-depletion studies used surprisingly small sample sizes, with an average *n* of 27 per condition (inter-quartile range between *n*s of 17 and 31), yielding a power ranging from 0.31 to 0.69, lower than the recommended 0.80. These small studies tended to report larger ego-depletion effect sizes than those that used more adequate sample sizes. After statistically controlling for small study effects, Carter and McCullough [16] suggested that the true ego-depletion effect may be smaller than typically reported in the literature (though some disagree with the use of some of these meta-analytic techniques; [19]). Furthermore, although small study effects can have a variety of causes, Carter and McCullough eliminated some alternative explanations and concluded that it was likely due to publication bias. If the true effect is smaller than researchers expect it to be, it will be difficult to detect unless a larger than typical sample size is used.

#### Potential p-hacking

Given the known publication bias against null and negative findings, another possible reason for reexamining the ego-depletion effect is that pressure to reach statistical significance (i.e., *p*-value less than .05) may cause researchers to engage in questionable research practices [20] that exploit the flexibility in data collection and data analysis (sometimes called “researcher degrees of freedom”; [21]). This flexibility comes in the form of, but is not limited to, conducting analyses throughout data collection in search of statistical significance (e.g., data peeking), controlling for covariates (e.g., gender) without compelling justification, and hypothesizing after the results are known (HARKing; [22]). Such p-hacking practices, however, lead to inflated effect sizes and an increase in false-positives.

These practices appear to be quite widespread in psychology [20, 23], and there is some evidence that such practices may have occurred in the ego-depletion literature. For example, significant findings were occasionally obtained by using one-tailed analyses (e.g., [24]) or by inappropriate rounding of *p*-values (e.g., reporting a *p*-value of .054 as *p* < .05; [25–27]). Also, despite the lack of theoretical justifications for doing so, some studies control for covariates (e.g., frustration experienced during the initial task; [28]) only after the typical analysis is not significant, or remove an entire group (e.g., women; [29]) post-hoc to show a significant ego-depletion effect.

#### Lack of clear understanding of potential moderator variables affecting the ego-depletion effect

In addition to these methodological issues, another important reason for reexamining the ego-depletion effect is that, despite the popularity of the phenomenon in both the scientific community and the media, there is very little understanding of robust and systematic moderator variables, such as task characteristics and individual differences, that shed light on the circumstances under which the ego-depletion effect is attenuated or intensified. In other words, ego-depletion may be easier to detect with particular types of self-control tasks or with particular types of participants. This is the issue we tackle most directly in the present study.

Regarding task characteristics, there is some evidence that task difficulty may moderate the ego-depletion effect, although previous studies addressing this possibility have yielded inconsistent results [5, 30]. In addition, it is possible that the ego-depletion effect may be stronger for longer outcome tasks as they might require more self-control and thus be more likely to lead to ego-depletion. Similarly, individual differences in motivation and effort might moderate the effect. For example, participants who are especially motivated to perform the depletion task might be in a greater state of ego-depletion, and so considering this moderator variable might improve the likelihood of detecting the effect.

Another potential moderator of the magnitude of the ego-depletion effect is variation in how closely participants follow the instructions during the depletion phase. For example, in the widely used White Bear task (e.g., [25–27]), participants in the depletion condition are instructed to write down their thoughts while not thinking of a white bear. Assuming that participants are following instructions, this task should require them to engage in self-control. However, even though condition differences in effort have been reported (e.g., [25]), given the nature of the task, it is difficult to objectively measure and verify what participants are actually doing during the task (i.e., suppressing specific thoughts in the depletion condition compared to not suppressing any thoughts at all in the control condition). Similarly, in the widely used video-viewing attention control task, participants view a 6-min video in which video footage of a woman being interviewed appears in one portion of the screen, while words appear one at a time in another portion of the screen (e.g., [5, 31–33]). Those in the depletion condition are instructed to ignore the words and instead focus all of their attention on the woman being interviewed (presumably taxing self-control). Participants in the control condition receive no mention of the words whatsoever even though they are a highly salient feature of the video. Therefore, some control participants are likely to purposely remember them if they think they will be tested on them after the video, while others may exert effort to ignore them. To our knowledge, no ego-depletion studies using this task have explicitly measured whether participants in the depletion condition actually followed instructions or whether participants in the control condition ignored the words, memorized the words, or viewed the video passively. Taken together, these variations in strategies during the depletion task could blur the distinction between the control and depletion conditions.

#### Present Study

Our group’s original interest in the ego-depletion effect was to examine, from the perspective of individual differences, to what extent a construct popular in social psychology, self-control, and a construct popular in cognitive psychology, executive functions (e.g., [34–35]), are related to each other. The ego-depletion effect seems to be a good candidate measure of self-control in examining the relationship between self-control and executive functions. However, given the recent controversy regarding the ego-depletion effect summarized in the previous section, we decided to first investigate whether the ego-depletion effect is reliable and whether there are any circumstances (task characteristics or individual differences variables) in which the ego-depletion effect can be amplified or attenuated.

In this study, for the depletion task, we used the actual video material and verbatim instructions from the popular video-viewing attention control task employed by prior researchers. For the outcome task, we used a complex working memory span task known as the operation span task (OSPAN; [36]). We selected these particular tasks because they maximize the chances of demonstrating the ego-depletion effect for the following reasons: a) the video-viewing attention control task is one of the most widely used tasks and has consistently been shown to induce ego-depletion in prior research (e.g., [5, 31–33]); b) the OSPAN task places heavy demand on executive attention and hence should tax self-control resources (e.g., [37]); c) this particular combination of tasks has produced medium to large effect sizes in prior studies [5, 38]; d) these tasks are matched in terms of stimulus modality (i.e., both tasks utilize words), which has been shown to amplify the ego-depletion effect [38]. We planned to test a total of 200 participants, with 100 participants in each condition, yielding a sample size that is approximately 4 times larger than typical ego-depletion studies.

This experimental set-up allowed us to explicitly avoid issues of past research in this domain noted earlier. For example, to address the potential power issues and small-study effects, we tested a relatively large and adequately powered sample of participants. To reduce the influence of researcher degrees of freedom and to make our decisions transparent, we pre-registered our study hypotheses, detailed methods and procedures, and the complete data analysis plan (including exclusion criteria and the data-stopping rule) using the Open Science Framework repository (https://osf.io/). Our pre-registration was submitted prior to data collection, “frozen” to eliminate any possibility of post-hoc modification, and following manuscript publication, will be made accessible to the public. The same hypotheses, methods, and analyses reported in the pre-registration plan are reported in this final manuscript, with exploratory analyses or deviations from the planned methodology clearly marked as such.

Moreover, with this experimental set-up, we systematically examined how characteristics of the outcome task (task difficulty and time on task) as well as individual differences in motivation and effort impact the ego-depletion effect. We were also able to include measures to investigate what participants were actually doing during the depletion task (e.g., whether they were following instructions).

We had two primary goals in conducting this research. The first goal (represented by Hypothesis 1 below) was to attempt to replicate the ego-depletion effect as it is typically demonstrated in the existing literature but with a much larger sample size. Our pre-registration described Hypothesis 1b where we planned to investigate, with actual (rather than simulated) data, the extent to which the prevalence of small sample sizes contributes to “false positive” results. Specifically, we tested how sample size might affect estimates of effect sizes and frequency of false positive results by conducting bootstrap resampling analyses with group sizes ranging from 10 to 50. However, the results of these analyses showed nothing more than typical results from simulated data. Therefore, they are not discussed further and are instead reported in S1 Appendix.

The second goal of this research (represented by Hypotheses 2a and 2b below) was to identify any moderator variables that may impact the magnitude of ego-depletion effects. In particular, we examined the potential influence of task difficulty and time on task on the outcome task (Hypothesis 2a) as well as the potential influence of participants’ motivation and effort during the experimental session (Hypothesis 2b). In other words, we examined under what circumstances and for whom the ego-depletion effect might be most robust and reliable.

We also hypothesized that individual differences in participants’ adherence to task instructions during the depletion task may moderate the magnitude of the ego-depletion effect. For example, given the ambiguity of the task instructions and the odd nature of the content in the attention control video, participants might differ in their level of compliance with our intended treatment: some depletion participants might follow instructions (thus engaging self-control as intended) whereas others might view the video more passively, and some control participants might engage in self-control while watching the video whereas others might view the video passively. The present study therefore serves as a first step toward specifying such moderating influences of task (2a) and individual (2b) characteristics that might be operating in the ego-depletion paradigm.

### Hypotheses

In this study we set out to test—either support or refute—the following hypotheses, corresponding to the main study goals outlined above:

**1. Group-level ego-depletion effect.** Participants in the Depletion Condition will perform worse on a subsequent self-control task compared to participants in the Control Condition who perform no initial act of self-control.

**2a. Moderating effects of task characteristics.** The magnitude of the ego-depletion effect will vary with task difficulty and time on task.

**2b. Moderating effects of individual differences.** Certain participant characteristics (e.g., high motivation on the depletion task, high effort on the depletion task, high on the extent to which they dutifully ignored the words on the screen during the ego-depletion task) will be associated with greater ego-depletion.

### Pre-Registration

We pre-registered the method and planned analyses on the Open Science Framework. This was done on October 1, 2014, prior to any data collection, and can be found at https://osf.io/cifn3/?view_only=0c7de573120f4cab9b52e7ec2b2acd2d.

## Method

### Participants

Two hundred participants (78 male, 122 female) were recruited from the human subject pool of the Department of Psychology and Neuroscience at the University of Colorado Boulder and received partial course credit for their participation. This research was approved by the University of Colorado Boulder Institutional Review Board, and participants provided written informed consent. Only native English speakers were eligible to participate due to the use of the OSPAN, which involved the active maintenance of verbal stimuli (words). Participants were randomly assigned to the Depletion Condition or Control Condition. Prior to the study, the first 50 participants were randomly assigned to conditions, and we repeated this for each subsequent 50 participants.

Data collection was terminated after 200 usable, complete datasets. This sample size was selected because it is nearly four times the typical sample size used in ego-depletion studies (*n* = 27 per condition; [6]). Although the reported average effect size in Hagger et al.’s [6] meta-analysis is 0.62 (Cohen’s *d*), Carter and McCullough [16] suggested that the actual effect size might be considerably smaller. With our relatively large sample, we were able to detect effects of at least 0.40 (Cohen’s *d*) at 80% power.

Our a priori exclusion criteria were as follows:

- Leaving the experiment session before completing the tasks
- Performing the OSPAN with worse than 80% accuracy on the equation verification task
- Correctly guessing at least one of the hypotheses of the study
- Admitting to not answering honestly to questions in the experiment
- Being a non-native English speaker

These exclusion criteria applied to two participants. One participant failed to complete the final questions due to an experimenter error, and one participant performed the OSPAN with only 76% accuracy on the equation verification task. None of the participants correctly guessed a hypothesis, admitted to not answering questions honestly, or were non-native English speakers. Therefore, we collected data from a total of 202 participants leaving us with 200 usable datasets (100 in each condition).

### Materials and Procedure

Participants first completed an initial ego-depletion or control task (video-viewing attention control task) followed by an outcome task (OSPAN). All participants then completed an assessment of their memory for the stimuli used in the video-viewing task so that we could objectively quantify the extent to which participants complied with their intended treatment (ignored or processed the words on the screen during the video-viewing task; participants did not know in advance that they would be tested on their memory of the words in the video later). At the very end, they answered a series of additional questions about strategies they used during the video-viewing task.

Each participant was tested individually in a single session lasting approximately 30 minutes. The experimenter stayed in the room with the participant during the entire course of the session. (The ego-depletion effect appears to be uninfluenced by whether the experimenter stays in the room or leaves during the video task; B. Schmeichel, personal communication, September 2014.) To prevent the influence of experimenter expectations, all experimenters conducted both conditions and read verbatim from a script. Moreover, the experimenters were unaware of the hypotheses and uneducated on the ego-depletion phenomenon, and all debriefing forms were sealed in envelopes to remain hidden from experimenters. When the experimenters were interviewed after the completion of the study, none of their ideas about the study goals matched the actual study goals and hypotheses we were testing in this study.

#### Ego-depletion task

The video-viewing task used in this study is one of the most widely used ego-depletion tasks in the ego-depletion literature. We used the exact task developed by its original author [32] including the verbatim script provided by the author (B. Schmeichel, personal communication, September 2014). In this task, participants viewed a silent 6-min video of a woman being interviewed (downloaded from http://psy.fsu.edu/~baumeisterticelab/egodepletion.html). During the video, 36 common one-syllable words appeared one at a time in black font against a white background at the lower right of the screen for 10 seconds each.

All instructions were read aloud, verbatim by the experimenter from the script, and the participants followed along on the computer screen.

Participants in the *Depletion Condition* received the following instructions:

This experiment investigates how people form impressions of others and how those impressions influence memory. So, I’m going to have you watch a short film clip that shows a woman being interviewed, but I’m going to turn the sound off so that you can only see the woman. Later I’ll have you answer some questions about your impressions of her. Since you won’t be able to hear what she’s saying you’ll have to base your impressions of her on her nonverbal behavior.

So, in addition to the woman being interviewed, you will also see some words on the bottom right of the screen. It is very important for the purposes of this experiment that you keep your attention focused only on the woman’s face and do not look down at the words that appear at the bottom of the screen. If you do accidentally look at the words, I want you to re-focus your attention on the woman as quickly as possible.

This task may be kind of difficult because the words take up a decent portion of the screen, but I want you to try hard to ignore those words and focus your attention only on the woman.

Later, you’ll have to answer some questions about your impressions of the woman based on her nonverbal behavior. When the clip ends, let me know. Remember, focus only on the woman and try to ignore the words.

Participants in the *Control Condition* received the following instructions:

This experiment investigates how people form impressions of others and how those impressions influence memory. So, I’m going to have you watch a short film clip that shows a woman being interviewed, but I’m going to turn the sound off so that you can only see the woman. Later I’ll have you answer some questions about your impressions of here. Since you won’t be able to hear what she’s saying you’ll have to base your impressions of her on her nonverbal behavior.

When the interview clip starts, I want you to watch it just as if you were sitting at home watching TV, even though the sound will be off. I don’t want you to worry about trying real hard to form an impression or anything. Just watch the clip and when it ends, let me know.

After they received the instructions, participants were asked, “How motivated are you to do well on this task?” (0: Not at all to 9: A lot).

While participants viewed the video, the experimenter remained in the room, but moved to a different corner of the room separated by a partition. After they completed the task, they notified the experimenter and were asked, “How much effort did you put into this task?” (0: None to 9: A lot).

#### Outcome task

The operation span task (OSPAN) [36], a commonly used measure of working memory capacity, was the main measure of ego-depletion in this study. This task was chosen because it requires participants to use executive control [37, 39], it allows variation of task difficulty in the form of different set sizes, and it has been used as the outcome task in ego-depletion studies in the past (e.g., [5]), including one that resulted in a particularly large effect size (Cohen’s *d* = 1.35, *N* = 38; [37]).

In this task, simple math equations (e.g., “3 + 1 = 6”) were presented one at a time. Participants were instructed to read the equation out loud and to say whether the result was true or false (in this example, “false”). Immediately upon answering, the experimenter responded on the keyboard with the “t” (for “true”) or “f” (for “false”) keys, and a single target word (e.g., “lamp”) appeared on the screen for 750 ms. This process then repeated. After a number of such equation-word pairs (ranging from 2 to 6 per trial), participants were instructed to recall out loud all the words of that trial in correct serial order, and the experimenter recorded all the responses on the scoring sheet. The dependent measure was the proportion of words recalled in correct serial order, which is a commonly used index for this type of complex working memory span measures (e.g., [40]). To examine whether the ego-depletion effect depends on task difficulty, we varied task difficulty by using two trials of each set size ranging from 2 to 6 memoranda. To evaluate the influence of time on task, trials were presented in two blocks each with ascending set size, allowing for comparison of the ego-depletion effect across the two task halves.

After receiving the instructions and practice trials (two trials of set size two) for this task, participants were asked, “How motivated are you to do well on this task?” (0: Not at all to 9: A lot), and after completing the task, participants were asked, “How much effort did you put into this task?” (0: None to 9: A lot).

#### Memory test for words in the video

To quantify how Control participants treated the words and to measure whether participants in the Depletion Condition followed instructions (i.e., by not looking at the words), we administered a “surprise” memory test after the OSPAN. Participants received the following instructions:

In the following memory test, you will be presented with words one at a time. Some of the words were in the video that you watched earlier. Some of the words are new. You have not seen them in any of the tasks today. Respond to each word with whether you remember seeing it in the video (OLD) or whether you do not think it was in the video (NEW). Then respond with how confident you are with your decision.

Eighteen of the 36 words from the video and 18 new words (not presented in the ego-depletion task or the OSPAN) were presented one at a time. The confidence ratings for each judgment were made on a 10-point scale (0: Not at all confident to 9: Very confident). Performance was analyzed with signal detection analysis, using the new-old judgments and confidence ratings. The dependent measure was d-prime (*d’*), which accounts for general tendencies in guessing on all items. For example, if the measure of test performance was the proportion of video words correctly recalled, and a participant responded “OLD” on all of the words, they would appear to have high performance, which should not be the case. Specifically, *d’* represents the ability to distinguish between new words and old words and can be intuitively understood as the difference between the hit rate (i.e., proportion of video words correctly responded with “OLD”) and false alarm rate (i.e., proportion of new words incorrectly responded with “OLD”). In fact, the simple subtraction of the hit rate and false alarm rate usually correlates very highly with *d’*, and this was true in this study, *r*(198) = 0.97, *p* < 0.001.

#### Additional measures

Finally, we included additional questions about the ego-depletion task at the end of the session to measure how participants interpreted the task and behaved during the task. Some were used to validate the memory test for words in the video by correlating memory scores with responses to the questions (e.g., we predicted that individuals who score high on the memory test will also indicate that they tried hard to remember the words from the video). Others were used as exclusionary criteria. They consisted of these items:

Used for validating the memory test:

- How well did you follow the instructions during the video task? (0: Not at all to 9: Entirely)
- Did you think we were going to ask you about the words later in the experiment? (0: Not at all to 9: Absolutely)
- When you saw the words in the video, how hard did you try to remember them? (0: Not at all to 9: A lot)
- When you saw the words in the video, how hard did you try to ignore the words? (0: Not at all to 9: A lot)

Used for later exclusion of participants:

- What did you think the purpose of this study was? (Free response)
- Did you answer these questions honestly? (Yes/No)

## Results

In accordance with our pre-registered analysis plan, we first tested the existence of the ego-depletion effect in the full sample (Hypothesis 1). Next, we explored task and individual characteristics that potentially maximize ego-depletion effects (Hypotheses 2a and 2b).

All statistical tests described below were conducted two-tailed with α = .05. Descriptive statistics for all variables are reported in Table 1.

**Table 1:** Descriptive statistics for all variables by condition.

Measures	*M* (*SD*)	Min	Max	Skewness	Kurtosis
**Depletion Condition**					
Video Task					
Motivation	7.13 (1.39)	4	9	-0.25	-0.67
+ Effort	6.59 (1.84)	1	9	-0.83	0.43
OSPAN					
Motivation	6.49 (1.28)	3	8	-0.53	-0.57
Effort	7.13 (1.03)	3	8	-1.19	1.31
Proportion Correct	.39 (.14)	.05	.80	0.24	0.16
Video Task Memory Test					
* Accuracy (*d’*)	0.54 (0.53)	-0.97	1.81	-0.18	-0.33
Video Task Questions					
* Q1	7.70 (1.26)	3	9	-1.17	1.36
* Q2	4.25 (3.39)	0	9	0.04	-1.55
* Q3	2.19 (2.50)	0	9	0.91	-0.40
* Q4	7.26 (2.48)	0	9	-1.56	1.50
**Control Condition**					
Video Task					
Motivation	7.17 (1.62)	1	9	-1.08	1.43
+ Effort	6.07 (1.89)	0	9	-0.67	0.13
OSPAN					
Motivation	6.77 (1.18)	2	8	-1.35	3.35
Effort	7.06 (1.11)	4	8	-1.00	-0.09
Proportion Correct	.36 (.13)	.10	.62	-0.05	-0.70
Video Task Memory Test					
* Accuracy (*d’*)	1.59 (0.91)	-0.32	3.51	0.13	-0.52
Video Task Questions					
* Q1	7.13 (1.72)	1	9	-0.87	0.50
* Q2	6.75 (2.63)	0	9	-1.07	0.03
* Q3	4.69 (2.67)	0	9	-0.29	-0.80
* Q4	3.06 (2.49)	0	9	0.50	-0.67

*Note*. Q1: “How well did you follow the instructions during the video task?”, Q2: “Did you think we were going to ask you about the words later in the experiment?”, Q3: “When you saw the words in the video, how hard did you try to remember them?”, Q4: “When you saw the words in the video, how hard did you try to ignore them?“

* Indicates condition mean differences (*p* < .05).

+ Indicates condition mean differences (*p* < .10).

### Hypothesis 1: Group-Level Ego-Depletion Effect

**Fig 1:**
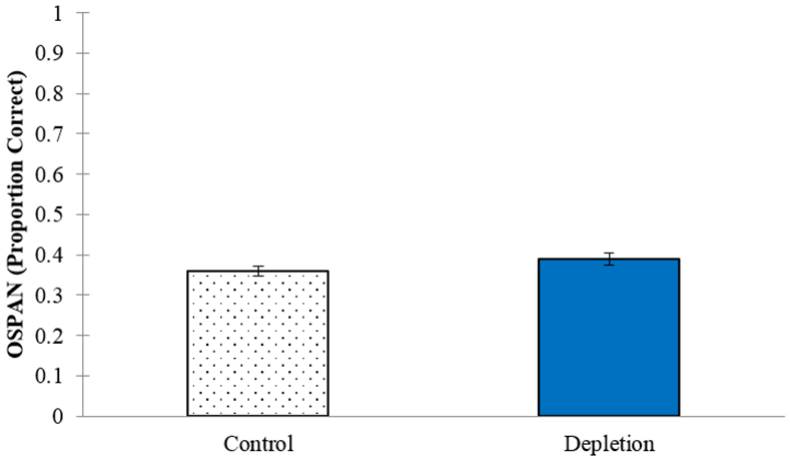
OSPAN performance between conditions. Error bars represent standard error of the mean (SEM).

Our main measure of ego-depletion was performance on the OSPAN (Cronbach’s α = .66), with an ego-depletion effect being reflected by lower performance in the Depletion Condition compared to the Control Condition. In line with how the ego-depletion effect is typically analyzed in the literature, we conducted an independent samples *t*-test. However, contrary to the predictions of the hypothesis, we observed no effect of condition, *t*(198) = 1.46, *p* = .146, *d* = 0.22, with participants in the Depletion Condition (*M* = .39, *SD* = .14) even showing slightly better performance than those in the Control Condition (*M* = .36, *SD* = .13; see Fig 1). However, given that a lack of statistical significance indicated by probability testing cannot speak to evidence for or against a null hypothesis, we also computed the Bayes factor associated with this *t*-value with the online calculator found at http://pcl.missouri.edu/bayesfactor and described by Rouder, Speckman, Sun, Morey, and Iverson [41], which indicated the data were in favor of the null hypothesis (scaled JZS Bayes factor = 2.40, scale *r* = .707; this analysis was not pre-registered).

### Hypothesis 2a: Moderating Effects of Task Characteristics

**Fig 2:**
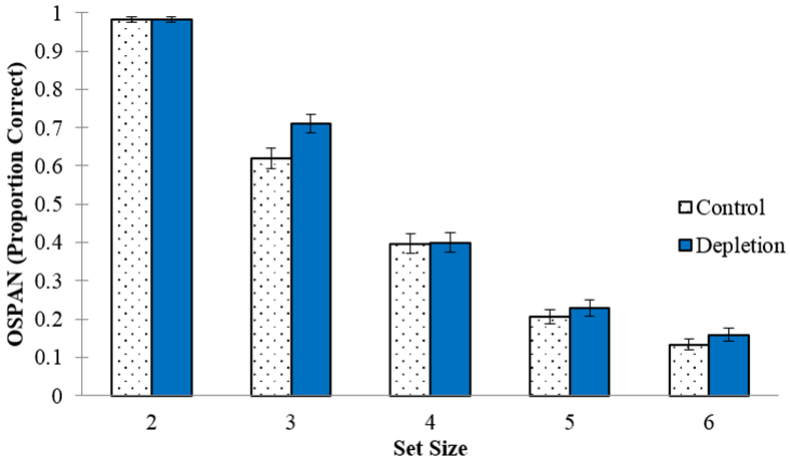
OSPAN performance between conditions at different set sizes. Error bars represent SEM.

We examined two characteristics of the OSPAN task that could potentially moderate the ego-depletion effect: task difficulty and time on task. Given the overall rather low performance on the OSPAN task (cf. Table 1), it is possible that the ego-depletion effect occurs only at particularly easy or particularly challenging levels of the OSPAN (i.e., specific set sizes). We explored task difficulty by running a mixed analysis of variance (ANOVA) on OSPAN performance with condition (between-subjects) and set size (within-subjects) as the independent variables. Unsurprisingly, task difficulty had a large effect, *F*(4, 796) = 762.82, *p* < .001, η_p_^2^ = .79, with increasing set sizes yielding performance decreases as indicated by a significant linear effect of task difficulty, *F*(1, 199) = 4439.81, *p* < .001, η_p_^2^ = .96. A marginally significant interaction between condition and set size, *F*(4, 792) = 2.13, *p* = .076, η_p_^2^ = .01, indicated that Depletion participants outperformed Control participants on set size 3, *t*(198) = 2.42, *p* = .016, though there was no reason to expect this and so the effect was likely spurious. There were no condition differences for any other set size (see Fig 2). The linear effect of task difficulty also did not interact with condition, *F*(1, 198) = 0.08, *p* = .780, η_p_^2^ < .01.

**Fig 3:**
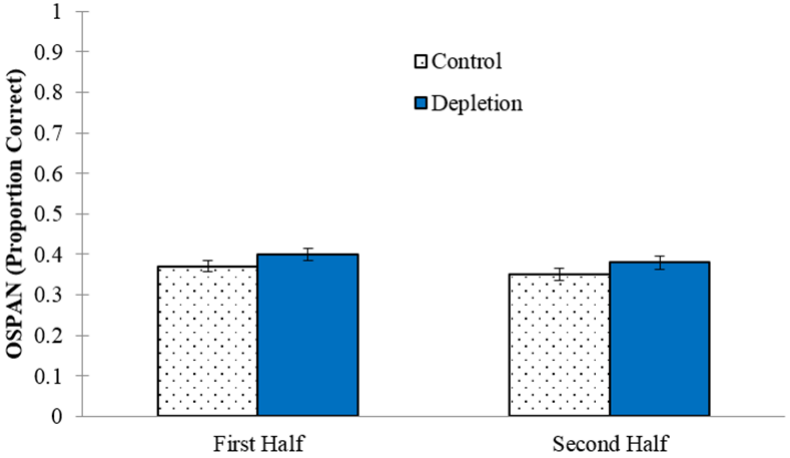
Condition differences on OSPAN performance between the first and second half of the task. Error bars represent SEM.

Regarding time on task, it is possible that ego-depletion effects only occurred immediately after depletion took place, or, on the contrary, required some time to unfold. Therefore, we compared performance in the first half of the task to performance in the second half. On average, OSPAN performance was better during the first half of the task (*M* = .39, *SD* = .14) compared to the second half (*M* = .37, *SD* = .15) with marginal significance, *t*(199) = 1.97, *p* = .051, η_p_^2^ = .02. However, a mixed ANOVA on OSPAN performance with condition (between-subjects) and task half (within-subjects) as the independent variables revealed that the effect of time on task did not differ by condition, *F*(1, 198) = 0.092, *p* = .762, η_p_^2^ < .01 (see Fig 3). Taken together, these results show that even after considering certain task characteristics that we might reasonably suspect the ego-depletion effect to depend on, we still see no evidence of ego-depletion.

### Hypothesis 2b: Moderating Effects of Individual Differences

Next, we examined individual differences variables that might influence the strength of the ego-depletion effect (and, as a consequence, our ability to detect it). To accomplish this, we analyzed responses to our measures of how motivated participants were to perform and how effortful they found the video and the OSPAN tasks. In addition, we investigated the strategies employed during the video task by analyzing performance on the video task memory test and the video task questions. Condition differences on these measures are presented in Table 1. Zero-order correlations of these variables and OSPAN performance are presented in Table 2.

**Table 2:** Zero-order correlations with OSPAN performance by condition.

	Total	Depletion Condition	Control Condition
Video Task			
Motivation	-0.084	0.038	-0.197*
Effort	-0.039	-0.074	-0.032
OSPAN			
Motivation	-0.057	-0.031	-0.063
Effort	0.045	0.102	-0.020
Video Task Memory Test			
Accuracy (*d’*)	-0.029	0.102	0.001
Video Task Questions			
Q1	0.067	-0.007	0.096
Q2	-0.120+	-0.100	-0.070
Q3	-0.027	-0.034	0.077
Q4	0.017	-0.068	-0.063

* *p* < .05.

+ *p* < .10.

#### Motivation and effort

We found no differences between groups regarding motivation before beginning the video task (after the instructions). Participants in the Depletion Condition reported being as motivated to perform the task (*M* = 7.13, *SD* = 1.39) as participants in the Control Condition (*M* = 7.17, *SD* = 1.62), *t*(198) = 0.19, *p* = .852, *d* = 0.03. If the video task demands more self-control in the Depletion Condition than in the Control Condition, effort ratings should be higher in the first group. That was precisely the case (see Table 1), with Depletion participants rating the video task as more effortful (*M* = 6.59, *SD* = 1.84) than Control participants (*M* = 6.07, *SD* = 1.89). The group difference, however, was only marginally significant, *t*(198) = 1.97, *p* = .050, *d* = 0.28. Regarding the OSPAN, there were neither differences between conditions in motivation, *t*(198) = 1.61, *p* = .110, *d* = 0.23, nor effort, *t*(198) = 0.46, *p* = .644, *d* = 0.07.

Even though we observed only little group-level differences in motivation and effort, it is still possible that these measures would interact with the strength of the ego-depletion effect. For example, participants who rated effort during the video task particularly high, may have exerted more self-control and, thus, show larger ego-depletion effects. Therefore, in a series of multiple regression analyses, we included condition, one of the individual differences variables, and their interaction, and none of these variables interacted with condition to predict OSPAN performance (*p*s > .134). In addition to this pre-registered procedure, we also entered all of the variables simultaneously in a single multiple regression model and the interactions remained non-significant (*p*s > .161).

#### Strategies during the video task

**Fig 4:**
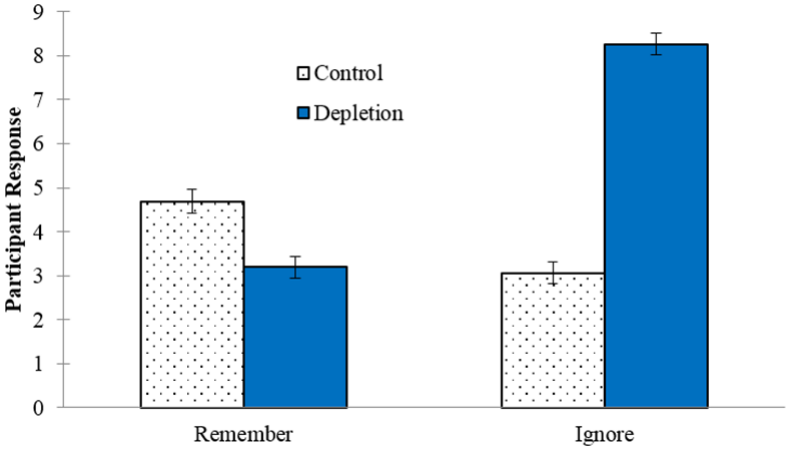
Condition differences in how hard participants reported they tried remembering and ignoring the words during the video task. Error bars represent SEM.

To explore what participants actually did during the video task, and in the Control Condition in particular, we analyzed performance on the video task memory test, which we assume to indicate whether participants ignored the words during the video or instead tried to memorize these words. This serves as a manipulation check to ensure that Depletion participants actually ignored the words, and to check that Control conditions did not actively attempt to memorize the words. Participants in the Depletion Condition were explicitly instructed to ignore the words on the screen, so if they followed this instruction, they should show a lower recognition performance. That was precisely the case, with participants in the Depletion Condition showing lower performance (*M* = 0.54, *SD* = 0.53) than those in the Control Condition (*M* = 1.59, *SD* = 0.91), *t*(198) = 9.96, *p* < .001, *d* = 1.41 (see Table 1). This is in line with the condition differences in their self-reports of how hard they tried to remember the words on the screen, *t*(198) = 6.85, *p* < .001, *d* = 0.97, and of how hard they tried to ignore the words on the screen, *t*(198) = 11.94, *p* < .001, *d* = 1.69 (see Fig 4).

**Fig 5:**
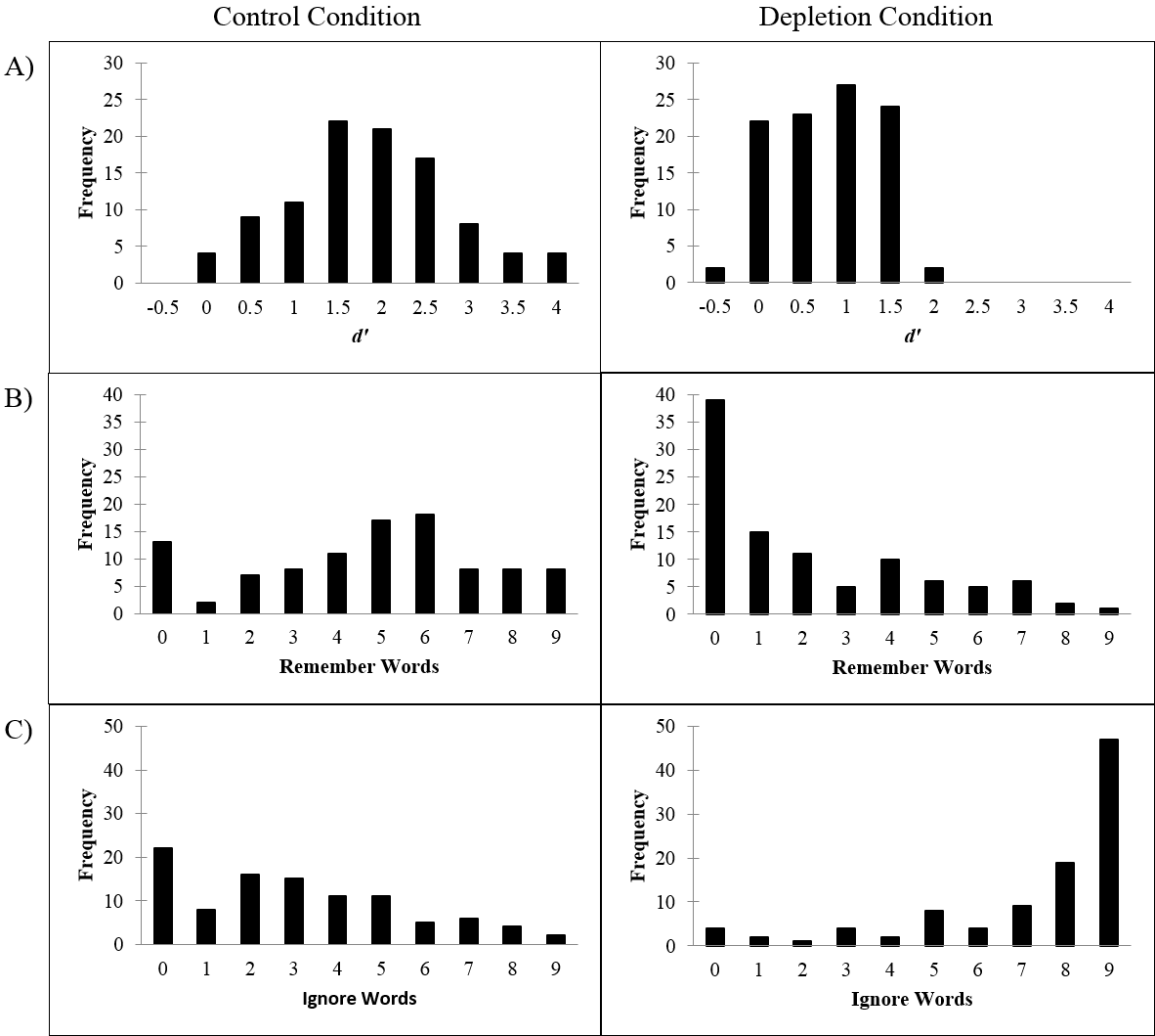
Histograms depicting strategies used during the video task. Higher numbers on the x-axes reflect better memory performance (A), greater effort to remember the words (B), and greater effort to ignore the words (C).

To further evaluate the strategies participants implemented during the video task, we inspected response distributions by condition (Fig 5). The Control participants varied more widely in the strategies they implemented during the video task as indicated by a less skewed distribution of responses in this condition (trying to remember the words: -0.29; trying to ignore the words: 0.50) compared to Depletion participants (0.91; -1.56). In other words, a substantial number of the Depletion participants reported trying hard to ignore the words and not trying hard at all to remember them. These responses were more evenly distributed among Control participants. This is not surprising because Control participants did not receive any specific instructions regarding the words in the video task (akin to how the task is typically administered). What is surprising is that the majority of Depletion participants (61%) reported exerting some level of effort (at least 1 out of 9) trying to remember the words despite being instructed to ignore them. The distribution of the scores for the more objective memory measure (*d’*) converges on these subjective ratings obtained from participants (see Fig 5A).

This information is useful because Control participants who reported ignoring the words engaged in self-control, and Depletion participants who reported not having tried ignoring the words did not engage in self-control, thereby potentially attenuating the ego-depletion effect. For example, despite the absence of an ego-depletion effect across all participants, evidence for ego-depletion could emerge between Depletion participants who tried especially hard to ignore the words and Control participants who did not try especially hard to ignore the words. In addition to the pre-registered analyses, we therefore tested this possibility first by focusing on participant reports of how hard they tried to ignore the words.

For this purpose, we reverse-coded responses to this question for Control participants so that higher numbers indicated less effort ignoring the words. We tested a multiple regression model predicting OSPAN performance with condition, how hard participants tried ignoring words (reverse coded for Control participants), and their interaction. Neither of the main effects nor their interaction, *F*(1, 196) = 0.86, *p* = .362, η_p_^2^ < .01, were significant, suggesting that conditions did not differ on OSPAN performance even when controlling for differences in how hard participants tried to ignore the words. We obtained similar results for how hard participants tried to remember the words (reverse-coded for all participants), *F*(1, 196) = 0.58, *p* = .454, η_p_^2^ < .01. Taken together, this evidence suggests that strategies during the video task did not moderate condition differences in OSPAN performance, and therefore, cannot explain the absence of the ego-depletion effect in our data, even though participants differed substantially in the extent to which they followed the instructions.

## Discussion

Although the ego-depletion effect is reported to be strong, reliable, and highly replicated (e.g., [6]), a few failed replication attempts have recently been published [12–13]. Additionally, a recent meta-analysis that used stricter inclusion criteria and as many unpublished studies as possible found little evidence for the ego-depletion effect [42]. As a result, some researchers (e.g., [16]) have recently issued a call for “determining whether truly convincing empirical support for the foundational finding of the model exists” (p. 2) for the ego-depletion effect.

The present study was conducted in response to this call and set out to examine the robustness and reliability of the ego-depletion effect with one particular combination of tasks, as well as task characteristics and individual differences that may moderate it. We used a larger than typical sample size (*N* = 200) to give us the ability to detect an effect smaller than the previously reported average effect size (Cohen’s *d* of 0.62) that may have been inflated due to publication bias, small-study effects, and potential p-hacking. Moreover, we pre-registered our hypotheses, methods, and planned analyses to prevent the influence of researcher degrees of freedom.

Despite these efforts, we found no evidence of ego-depletion: participants in the Depletion Condition did not perform differently from participants in the Control Condition on the outcome task, contrary to the ego-depletion hypothesis. We analyzed our data in a manner consistent with typical ego-depletion studies (i.e., a between-subjects independent *t*-test), and also explored several possible moderator variables in an attempt to uncover ego-depletion effects, but found no evidence for self-control differences between conditions.

A unique feature of this study was that we measured what participants were actually doing during the video task. Although participants in the Depletion Condition are instructed to ignore the words, prior studies have not measured whether participants were actually following instructions. This is important because participant strategies are likely to vary. There will likely be participants in the Depletion Condition who do not follow instructions, and the strategies are likely to vary widely among Control participants who do not receive instructions regarding the words. Both were the case in the present study. However, we found no evidence for ego-depletion effects even after controlling for what participants were actually doing during the depletion task.

### Difficulty Detecting Ego-Depletion

Here we consider four (of many) possible explanations for why we failed to detect evidence for ego-depletion in the present study, despite our best efforts. First, as Carter and McCullough [16] suggested, reported effect sizes in ego-depletion studies may often be inflated. Although we used a larger than typical sample size (*N* = 200) in the present study, we only had sufficient (80%) power to detect an effect of *d* = 0.40. If the true ego-depletion effect is smaller than *d* = 0.40, a larger sample size would be needed to detect it. However, we did use a sample size nearly four times what is typically used. Furthermore, if the present study had simply been underpowered to detect significant ego-depletion effects, we would at least expect to see a pattern consistent with the predicted direction of ego-depletion (i.e., with Depletion participants performing worse than Control participants on the outcome task). Instead, we observed the opposite pattern (i.e., with Depletion participants performing slightly but nonsignificantly better than Control participants on the outcome task).

Second, although none of the moderator variables we examined under Hypotheses 2a and 2b significantly moderated the magnitude of the ego-depletion effect in this study, there was clear evidence (see Fig 5) indicating that a substantial proportion of participants in the Control Condition actively engaged in memorization activities during the viewing of the video. Even though the instructions did not indicate any later “surprise” memory test, it was a natural thing for them to do, but active encoding of the words on the screen for potential future recall is clearly an attention-demanding task; in fact, as indicated in Fig 5A, some participants demonstrated an exceptionally high memory recall performance (e.g., *d’* > 3.0). In such a situation, the control task may have involved self-control as much as the Depletion Condition did. This could possibly explain past failures to replicate, though it is unlikely in the current study given our moderation analyses.

Third, ego-depletion may be influenced by moderator variables that we did not consider or measure. Although we explored some task characteristics and individual differences that may moderate ego-depletion, several other possibilities exist. For example, our experimenters were blind to the hypotheses of the present study, but perhaps experimenter expectations that Depletion participants will perform worse than Control participants on the outcome task are necessary to demonstrate ego-depletion. Another possibility is that the participants or testing environment of the present study somehow differed from that of typical ego-depletion research. Such possibilities should be tested in future studies to establish whether ego-depletion is only observed under certain circumstances or for certain types of individuals.

Fourth, it may be the case that participants in the Depletion Condition were in fact depleted, but we were unable to detect depletion with the OSPAN because that task reflects working memory capacity, not self-control. The inhibition of some impulse or other automatic process is not as obvious during the OSPAN as it is for other self-control tasks (e.g., inhibiting thoughts of a white bear during the white bear task). However, the use of a working memory measure to index self-control in sequential task paradigms [5, 38] is both conceptually and empirically supported. Conceptually, working memory is described similarly as self-control. It relies on aspects of goal maintenance supported by the executive function system [39], and it supports selective attention toward relevant stimuli and redirecting attention away from irrelevant distracting stimuli (e.g., [43, 44]). Empirically, working memory span performance has been shown to correlate with performance on several self-control tasks used in prior ego-depletion studies such as the Stroop task [31], suppressing facial expressions [2], delay discounting [45], overeating [46], and fluid intelligence [32; 35, 47–50]. Taken together, there is good justification for regarding the OSPAN task as a measure of self-control.

Of course, another possible explanation for why we failed to detect ego-depletion in the present study is that it is not a reliable psychological phenomenon. After controlling for small-study effects and publication bias, Carter and McCullough [16] suggested that the true effect of ego-depletion was not significantly different from zero. In other words, there may be a large body of null findings and significant negative findings challenging ego-depletion that have never been published, and the published evidence consistent with ego-depletion is simply an artifact of publication bias, small-study effects, and potential p-hacking.

### Recommendations for Future Research

Although the present study failed to detect evidence for ego-depletion with these particular tasks, more replication attempts are required before we can draw strong conclusions about the ego-depletion phenomenon, and these studies should explore the wide variety of tasks and task combinations as advocated by Carter et al. [42]. Future replication attempts should test large sample sizes, pre-register methods and planned analyses prior to data collection, and precisely measure potential moderator variables that might magnify or attenuate the ego-depletion effect.

The Registered Replication Reports (RRR) project, spearheaded by the editors of *Perspectives on Psychological Science*, is a strong model for coordinating and documenting replication attempts across multiple research groups. Several research labs agree to perform a direct replication of a particular, highly influential study, all with the same protocol. All details of the protocol are determined prior to data collection, and with the contribution of multiple labs, a large sample size is provided for analysis. Currently, an ego-depletion RRR is underway with 29 participating labs employing a protocol designed by Martin Hagger.

Future ego-depletion research would also benefit from exploring potential moderators (e.g., task characteristics, individual differences) that may help to explain why some studies demonstrate the effect while others do not. For example, a critical assumption of ego-depletion research is that participants in ego-depletion conditions are following instructions, and by following instructions they are engaging in self-control. In the present study, however, we found that a significant proportion of Depletion participants did not follow instructions to ignore the words on the screen during the video task, and a significant proportion of Control participant exerted effort to ignore the words, possibly taxing self-control. Although we did not find evidence of a depletion effect even when we controlled for strategy use across conditions, it is possible that such differences could attenuate condition-level differences, and thus partially explain the mixed pattern of findings in the literature.

Due to the difficulty we and other researchers have had in demonstrating the ego-depletion effect, it may also be time to address one major conceptual problem that we believe has plagued this field of research and hindered researchers’ efforts to conduct rigorous and compelling experimentation and theory development. Specifically, the problem is that researchers lack a consistent definition of self-control and poorly justify why some tasks rely on self-control and others do not. For example, in studies that demonstrated the ego-depletion effect on frustrating tasks, researchers argued that “overcoming frustration requires self-control” [26, p. 898]. However, in studies that demonstrated no condition differences on self-control performance when stress was manipulated, researchers argued that tasks can differ in stress without differing in self-regulatory effort [51]. There are even instances of the same task being used as the self-control task in one study and as the control task in another. For example, three-digit by three-digit multiplication has been used as both an outcome task, assumed to involve self-control (e.g., [33]), as well as a control task, assumed to not require self-control (e.g., [29]). There must be theoretical justification for why a particular task relies on self-control before the task is chosen for an ego-depletion experiment; otherwise, it is easier to hypothesize after the results are known that a particular task relied on self-control. Self-control is the basis for the ego-depletion hypothesis and the strength model of self-control and has yet to be sufficiently defined.

## Conclusion

We are far from concluding how robust and reliable the ego-depletion effect is. This area of research requires future replication attempts, and efforts to extend the strength model of self-control to other domains and manipulations should be secondary to determining a more accurate estimate of the ego-depletion effect. To reduce the likelihood of inflated reported effect sizes, we urge future research to use large sample sizes, consider moderator variables, define self-control and theoretically justify the use of particular tasks, and explicitly pre-register hypotheses, procedures, and planned analyses prior to data collection.

## Supporting Information

S1 Appendix Effects of Sample Sizes. (DOCX)

S1 Data (XLSX)
