# Supplementary material for: No Evidence of the Ego-Depletion Effect across Task Characteristics and Individual Differences: A Pre-Registered Study
Source: PLoS One. 2016 Feb 10;11(2):e0147770. doi: 10.1371/journal.pone.0147770 (PMC4749338; doi:10.1371/journal.pone.0147770)
Supplement: S1 Appendix — (DOCX) [file pone.0147770.s001.docx]

**Hypothesis 1b: Effects of Sample Sizes**

Ego-depletion effects are typically tested in sample sizes much smaller than in the present one (cf. [1]). Therefore, we examined how sample size affects the occurrence and size of the ego-depletion effect using a bootstrap resampling method [2]. For each of five hypothetical sample sizes (*n’*s per condition of 10, 20, 30, 40, and 50), we drew random subsamples (with replacement) to investigate whether significant ego-depletion effects (i.e., those meeting the *p* < .05 criterion) occurred more frequently in smaller sample sizes relative to larger sample sizes. For each of 1000 sampling iterations per sample size, we computed descriptive statistics (*M*, *SD*) and tested for group differences in OSPAN performance.

As summarized in Table A and Fig A, statistically significant ego-depletion effects in the expected direction (i.e., lower OSPAN performance in the Depletion compared to the Control Condition) occurred more frequently and were more variable in magnitude at smaller sample sizes. This is in spite of the fact that, at the level of overall means, subjects in the Depletion Condition performed slightly better than those in the Control Condition in this study (see Fig 1). For example, at the smallest condition size (*n* = 10), 306 of the 1000 iterations resulted in mean differences in the direction of the ego-depletion effect (i.e., better OSPAN performance in the Control Condition than in the Depletion Condition), with effect sizes ranging from *d* = 0.02 to *d* = 1.53 (*M* = 0.30), 3.60% of which reached statistical significance. As the sample size increased, the number of comparisons in the direction of the ego-depletion effect decreased along with the proportion of significant ego-depletion effects.

**Table A. Between-subjects outcome task performance from re-sampling comparisons.**

| *n* per Condition | % Iterations in which  Depletion < Control | Effect Size  *M* [min, max] | % *p* < .05 |
| --- | --- | --- | --- |
| 10 | 30.60% | 0.30 [0.02, 1.53] | 3.60% |
| 20 | 22.10% | 0.18 [0.01, 1.00] | 1.40% |
| 30 | 16.80% | 0.13 [0.01, 0.58] | 0.60% |
| 40 | 11.30% | 0.10 [0.01, 0.46] | 0.90% |
| 50 | 7.60% | 0.07 [0.00, 0.28] | 0.00% |

*Note.* The final two columns reflect effect sizes and % significant *p*-values for only those cases in which Depletion < Control. Effect size: Cohen’s *d*.

**
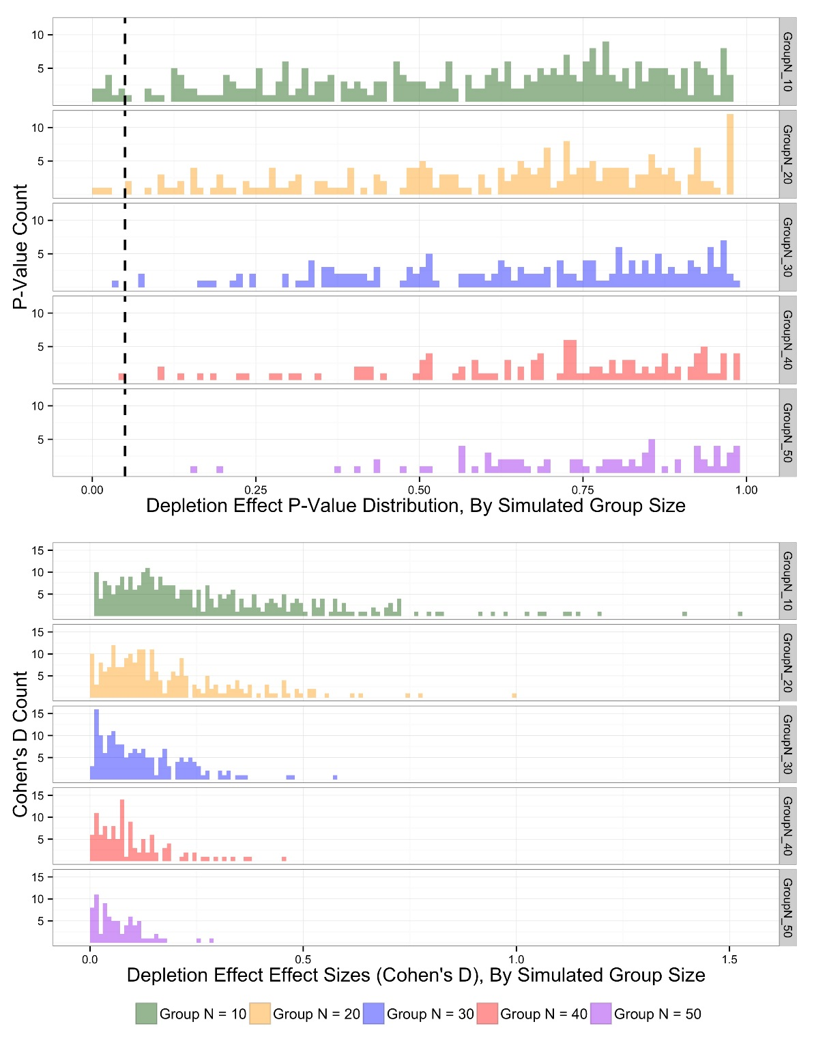
**

**Fig A. Distributions of p-values and effect sizes for ego-depletion effects (i.e., Depletion < Control) observed at varying sample sizes.** The dotted line reflects an alpha threshold of p < .05. Effect size: Cohen’s d.

**References**

1. Hagger MS, Wood C, Stiff C, Chatzisarantis NLD. Ego depletion and the strength model of self-control: A meta-analysis. Psychol Bull. 2010;136(4):495–525.

2. Efron B, Tibshirani RJ. An introduction to the bootstrap. New York: Chapman & Hall/CRC; 2003.
